# Rogue Waves: From Nonlinear Schrödinger Breather Solutions to Sea-Keeping Test

**DOI:** 10.1371/journal.pone.0054629

**Published:** 2013-02-06

**Authors:** Miguel Onorato, Davide Proment, Günther Clauss, Marco Klein

**Affiliations:** 1 Dipartimento di Fisica, Università degli Studi di Torino, Torino, Italy; 2 INFN, Sezione di Torino, Torino, Italy; 3 School of Mathematics, University of East Anglia, Norwich, United Kingdom; 4 Ocean Engineering Division, Technical University of Berlin, Berlin, Germany; Plymouth University, United Kingdom

## Abstract

Under suitable assumptions, the nonlinear dynamics of surface gravity waves can be modeled by the one-dimensional nonlinear Schrödinger equation. Besides traveling wave solutions like solitons, this model admits also breather solutions that are now considered as prototypes of rogue waves in ocean. We propose a novel technique to study the interaction between waves and ships/structures during extreme ocean conditions using such breather solutions. In particular, we discuss a state of the art sea-keeping test in a 90-meter long wave tank by creating a Peregrine breather solution hitting a scaled chemical tanker and we discuss its potential devastating effects on the ship.

## Introduction

Nowadays there is a general consensus on the existence of rogue waves in the ocean. During the last years many efforts in the community of physicists and oceanographers have been devoted to the understanding of their origin [1]. At the same time, engineers and naval architectures have been interested on the consequences of the impact of rogue waves on offshore structures and ships [2]. Up to know, scientists have identified a number of mechanisms of formation of rogue waves, the most obvious ones being the linear superposition of waves and the interaction of waves with currents [1]. More sophisticated mechanisms are based on the description of waves through the nonlinear dynamics of the Nonlinear Schrödinger (NLS) equation. Besides water waves, this equation can describe many different physical systems such as for example nonlinear optics, plasma waves and condensed matter [3]–[5]. In the oceanographic context, even though it is an approximation of the fully nonlinear equations, it contains the two basic ingredients of the surface wave dynamics: nonlinearity and dispersion. Since the late seventies, “exotic” NLS solutions, known as breathers, describing the modulation of a slightly perturbed wave have been discovered [6], [7] and more recently proposed as rogue wave prototypes [8], [9].

At the Technical University of Berlin, we have successfully produced breather solutions of the NLS and used for the first time in sea-keeping tests, opening up new perspectives in the methodology of examining offshore structures and ships against rogue waves. The solution considered in the present study is the Peregrine breather. It is characterized by an amplification factor of three [10], [11]: an unstable quasi-monochromatic wave with initial amplitude 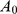 will lead during the evolution to a peak amplitude of 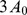
! In principle, the Peregrine solution can be built with arbitrary initial wave steepness, 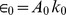, with 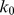 the wave number of the carrier wave; however, in nature, steepness hardly reaches values larger than 0.4 because of wave breaking [12]. While this represents a limitation in the theoretical description, it turns out that it is an advantage from an engineering perspective. Indeed, the possibility of creating a deterministic wave characterized by a maximum wave height for a given frequency allows one to study the impact of a breaking rogue wave on structures or ships, being in principle much more dangerous than non breaking ones (e.g. local loads, green water).

## Materials and Methods

### The Nonlinear Schrödinger Equation Model and the Breather Method

**Figure 1:**
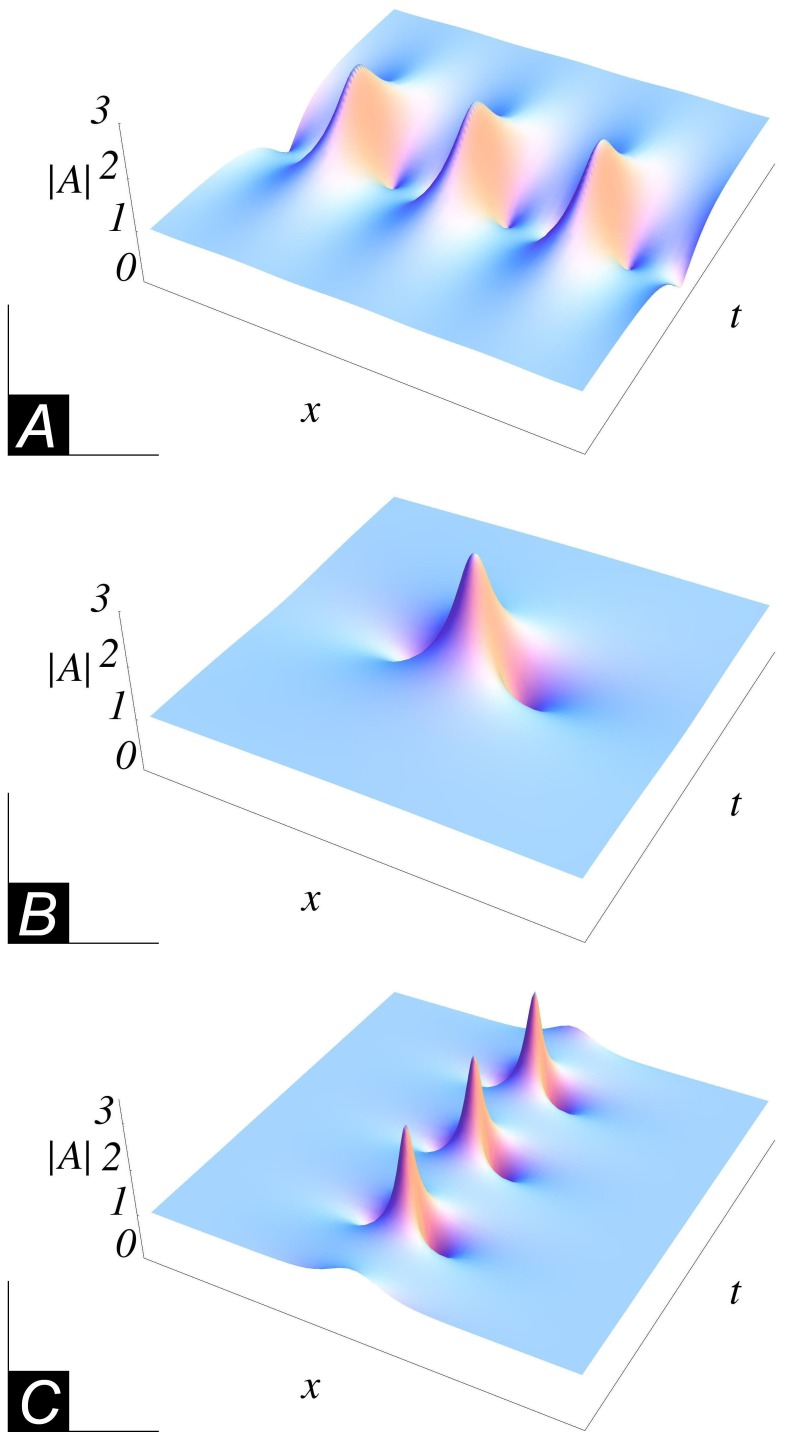
Absolute value of the complex envelope of exact analytical breather solutions of the NLS equation in nondimensional form. (A) The Akhmediev breather. (B) The Peregrine breather. (C) The Kuznetsov-Ma breather.

The dynamics of surface gravity waves is described by a set of nonlinear partial differential equations known as the Euler equations for water waves [13]. Being nonlinear, the equations are not easy to solve analytically and numerically; therefore, the physics of water waves is hidden by such difficulties. More than often, a simplification of the equations is needed in order to get some feeling on the interesting phenomena that may take place in the water wave dynamics. In order to address the problem of rogue waves, it has become standard to use a weakly nonlinear approach. In the present study we have considered the Nonlinear Schrödinger (NLS) equation as a simplified model of the original equations of motion. Such equation is formally derived from the Euler equation under the hypotheses that the waves are characterized by a small steepness and their spectrum is narrow banded [14]. In a coordinate system moving with the group velocity 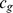, the equation results in [13]:
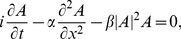
(1)where 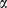 and 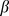 depend on the wave number of the carrier 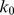 and on water depth 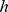 as



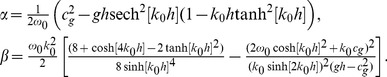
(2)Here 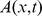 represents the complex wave envelope which is related to surface elevation 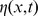 to the leading order in the following way:
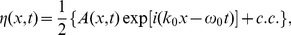
(3)with 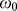 the angular frequency corresponding to the wave number 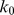, 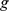 the gravitational constant, and 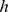 the water depth. The NLS equation presents a lots of advantages with respect to the original equations of motion: it is an integrable system and many of its analytical solutions can be explicitly written. After the discovery of the solitons, which are waves that travel without changing shape, it has been discovered that for deep water conditions, 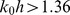, this system has breather solutions such as the Akhmediev [15], the Peregrine [16], and the Kuznetsov-Ma [6], [17] ones. Breathers solutions share the following property: they start (in space or time) with an almost constant background (corresponding to a quasi-monochromatic wave in terms of the surface elevation) and, during their evolution, the perturbation on the envelope grows reaching an amplitude that is much larger than the initial amplitude. The Akhmediev solution is periodic in space while the Kuznetsov-Ma one is periodic in time. The Peregrine solution has the peculiarity that appears only once and is not periodic in space or in time; moreover, it is characterized by an amplification factor of three, that is to say the maximum amplitude reached by the wave is three times the initial one. In Figure 1 we show such solutions in nondimensional form; note that they have different periodicity properties. All three solutions are rogue wave prototypes.

In the present study we have considered the Peregrine solution, even though extensive experimental work has been performed also with the Akhmediev and Kuznetsov-Ma solutions. The reason is that the Pereregrine solution has the maximal amplification factor. Hereafter we show the analytical form of the Peregrine solution:
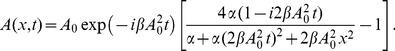
(4)


We underline that the Peregrine solution has been reproduced experimentally for the first time in [11], [18], [19]; in that case the solution was constructed in infinite water depth. In the present case, our aim is to reproduce the physical conditions that took place in the North Sea when the famous Draupner wave was recorded [20]; therefore, finite water depth effects are considered. The maximum amplification factor of the Peregrine solution is independent of the water depth; however, as the water depth decreases, the growth rate of the instability decreases as well. Indeed, experimental investigations on the influence of the formation process of breather solutions revealed that the mechanism of modulation instability leads to extraordinarily high waves also in intermediate water depths (as predicted by theory) but the distance that the group has to travel to reach the maximum wave height increases significantly with decreasing the water depth and approaches infinity (i.e. no modulation instability) for 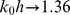 [21].

Our test are performed in the following way: first of all a target location (usually where the structure or ship are placed) is selected. This position corresponds to some distance from the wave-maker. The NLS Peregrine solution with a preselected steepness is then driven back in space from the target to the wave maker: this provides the desired time series furnished to the wave maker which will evolve into the rogue wave. Depending on the initial steepness, the wave will break or not at the point of its maximum height. In general we have found that waves of initial steepness less than 0.1 do not break. Larger steepness produces a breaking which is of the spilling type and then becomes of the plunging type as the initial wave steepness increases.

The present methodology of producing a breaking (or non breaking) rogue wave has strong advantages with respect to the linear superposition method usually employed in wave tanks to produce rogue waves. Indeed, the simplest way of producing a rogue wave in a wave tank consists in exploiting the quasi-linear dispersive properties of surface gravity waves. The idea is to generate first short waves and subsequently longer ones. The short waves are slower and are caught up by longer ones. If the experiment is well designed, at some distance from the wave maker, all waves generated are in phase and can be summed-up (linear superposition principle) to generate a large amplitude wave. Due to the fact that close to the focusing point, the steepness of the wave is not small, usually the focusing does not follow exactly the linear dynamics and some small nonlinear corrections are needed. Such method has been widely and successfully applied to generate rogue waves in wave tanks up to the breaking point [2], [22]. The disadvantage of the procedure is that the spectrum needed to generate a linear focusing is necessarily wide in frequency, so that many Fourier components must be summed up to produce the rogue wave. However, in sea-keeping tests, most of the time one is interested in studying the response of the ship to some particular frequencies (or wavelengths); for example one may wish to know if there exists a particular wavelength (with respect to the ship or fixed structure length) for which the impact of the rogue wave (characterized by that wavelength) can be more destructive or dangerous. Such kind of study cannot be accomplished within the linear focusing approach.

In this respect, the breather solution test method offers a unique opportunity: wave tank experiments can be performed with a preselected frequency allowing a direct analysis of the response of the ship. Moreover, because the breathers correspond to analytical solutions of the NLS equation, one may easily change the parameters of the initial conditions to produce rogue waves of the desired steepness (up to the breaking point). Last but not least, the shape of the resulting wave resembles very much the measurements of the rogue waves in the real ocean; this is of course a non-negligible aspect if one is interested in studying realistic sea state conditions.

### The Chemical Tanker Model

**Figure 2:**
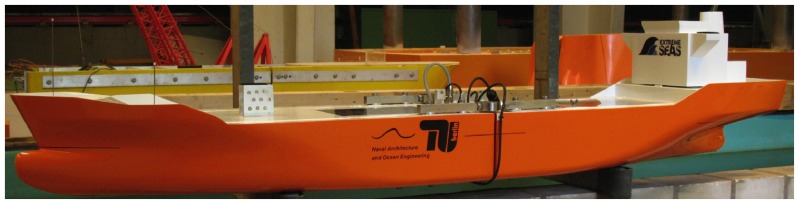
The chemical tanker model used in the experiments. It is made of fibreglass reinforced plastic and equipped with transducers. Model data are summarized in Table 1.

Our sea-keeping experiments are performed with a chemical tanker model shown in Figure 2, see also Table 1 for the model and full scale dimensions. In order to investigate the vertical bending moment, the model is subdivided into two segments amidships, being connected with three force transducers. Two transducers are installed on deck, one on each side, and one is mounted underneath the keel of the model. The force transducers register the longitudinal forces during the experiments. Based on the measured forces and the given geometric arrangement of the three force transducers the resulting vertical wave bending moment and the longitudinal forces are obtained. On this basis, the superimposed vertical wave bending moment resulting from the vertical and horizontal forces on the hull is determined. During the tests the model is towed with a flexible suspension system consisting of a triangular towing arrangement. The longitudinal motions are controlled with a spring in the front and a counter weight behind the model. That way, surge motions are restricted but heave and pitch motions as well as forces and moments remain unrestrained.

**Table 1:** The dimension of the model.

Chemical Tanker	Ship scale	Model scale
Length between perpendiculars	161.00 m	2.3 m
Length overall	170.0 m	2.429 m
Breath moulded	28.0 m	0.400 m
Depth moulded	13.0 m	0.186 m
Displacement	30707 t	89.5 kg
Midship Draft	9.0 m	0.129 m

The ship motions are recorded by an optical tracking system consisting of a seven by ten meter frame that carries four infrared cameras, which can be shifted parallel to the moving ship model. The system enables high precision, contact-free motion tracking over large distances by following the trajectories of infrared light emitting diodes mounted on the ship model. The surface elevations are measured at several positions with surface piercing resistance-type wave gauges installed on the towing carriage. A total amount of nine wave gauges are installed about the ship model position. The first one is 6.2 m in front of the forward perpendicular of the ship. A cluster of seven gauges, separated in 0.1 m intervals, is arranged around the forward perpendicular to investigate the spatial evolution of the waves at the bow. Furthermore one gauge is installed amidships. The above mentioned positions of the wave gauges are related to the stationary ship at the idle state knowing that the ship slightly oscillates in x-direction during the tests due to the restricted but not eliminated surge motion. For the tests the investigated wave sequences have also been generated and measured without the ship to ensure that radiated ship waves do not affect the measurements [21].

For the investigations of green water effects on deck resulting from the wave impact on the bow, two “green” water gauges are installed: the first one on the foredeck and the second one is approximately 17 m (full scale) behind on the weather deck. The maximum measurable head of water is approximately 13 m and 9 m (full scale), respectively.

### The Experiments

The tests have been conducted in the basin of the Ocean Engineering Division of Technical University of Berlin. The basin is 110 m long, with a measuring range of 90 m. The width is 8 m and the water depth is 1 m. On one side a fully computer controlled electrically driven piston type wave generator is installed. The control software features the generation of transient wave packages, deterministic irregular sea states with predefined characteristics as well as tailored critical wave sequences [23], [24]. As mentioned above, the experiments are performed in such a way that the target location (usually where the structure or ship are placed) and the initial steepness is defined at the beginning of the test campaign. In the context of the Peregrine solution the target location denotes the location of maximum amplification of the breather. The agreement between the theoretical location of freak wave occurrence and the observations in the tank was satisfactory as in most of the cases the freak wave event is nearby the given value. Tests with different frequency, amplitude and amplification factors have been performed. This has allowed us to gain a very deep knowledge on the nonlinear dynamics of such breathers.

The results presented in this paper are related to a systematic study on the impact of the Peregrine solution on the ship. As previously mentioned, the only free parameter in the Peregrine solution is the initial steepness, 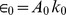, with 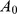 the initial amplitude of the perturbed monochromatic wave. The maximal amplification factor of the Peregrine solution is equal to 3, therefore the steepness of the rogue wave is 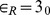. We have observed that for initial steepness larger than 0.1 the Peregrine solution starts breaking. Even though this is a failure of the theoretical description of the NLS that is not capable of describing the breaking of the wave, from an engineering point of view it represents an added value to the test procedure in the sense that allows one to study the impact of a breaking rogue wave on the structure. Note that forces and kinematics of a breaking wave can be much larger than a non-breaking one.

## Results and Discussion

**Figure 3:**
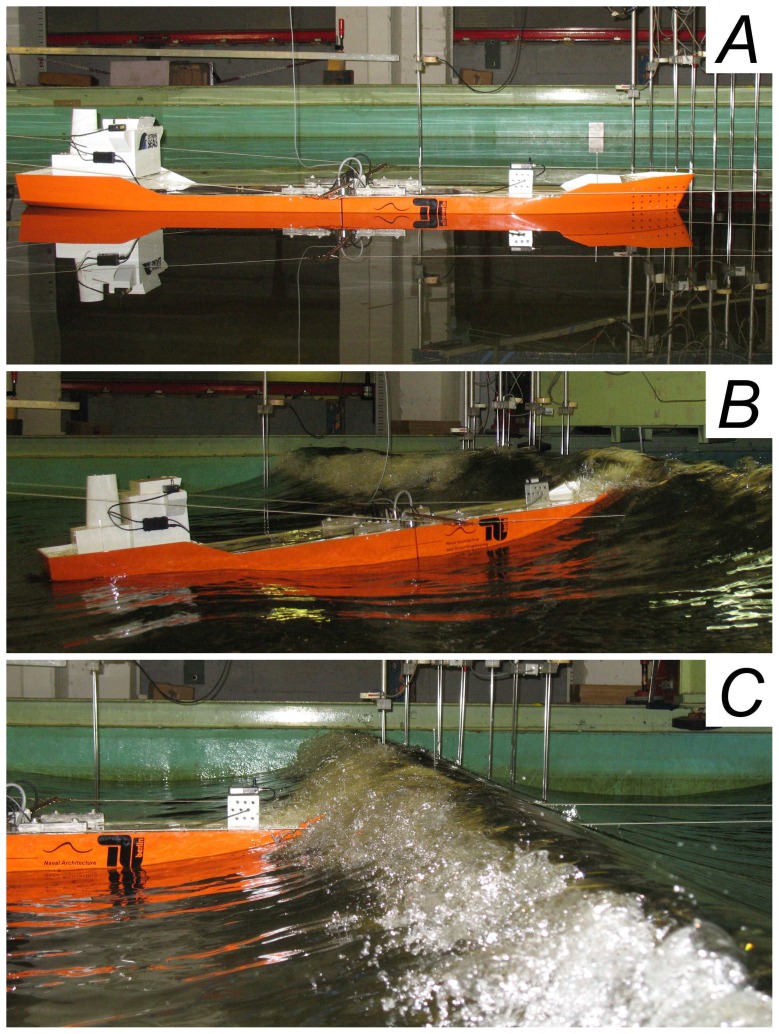
Sea-keeping tests using breather solutions of the NLS equation. (A) The chemical tanker used for the experimental tests. (B) The ship during the impact with a breaking Peregrine breather. (C) A detail of the impact to appreciate the green water on the deck.

We discuss here the case study of a Peregrine breather that has a height at full scale of 26 meters and wavelength of 240 m. Height and wavelength are very similar to the famous Draupner wave, measured in the North Sea [20]. The experiment is performed at a depth of 1 m and the wavelength of the carrier wave at the wave maker is 3.47 m, therefore 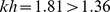; finite water depth are relevant but such conditions allows still for the modulational instability to take place. Figure 3 shows the sequence of the scaled breather impacting on the ship (for a complete video see Movie S1).

**Figure 4:**
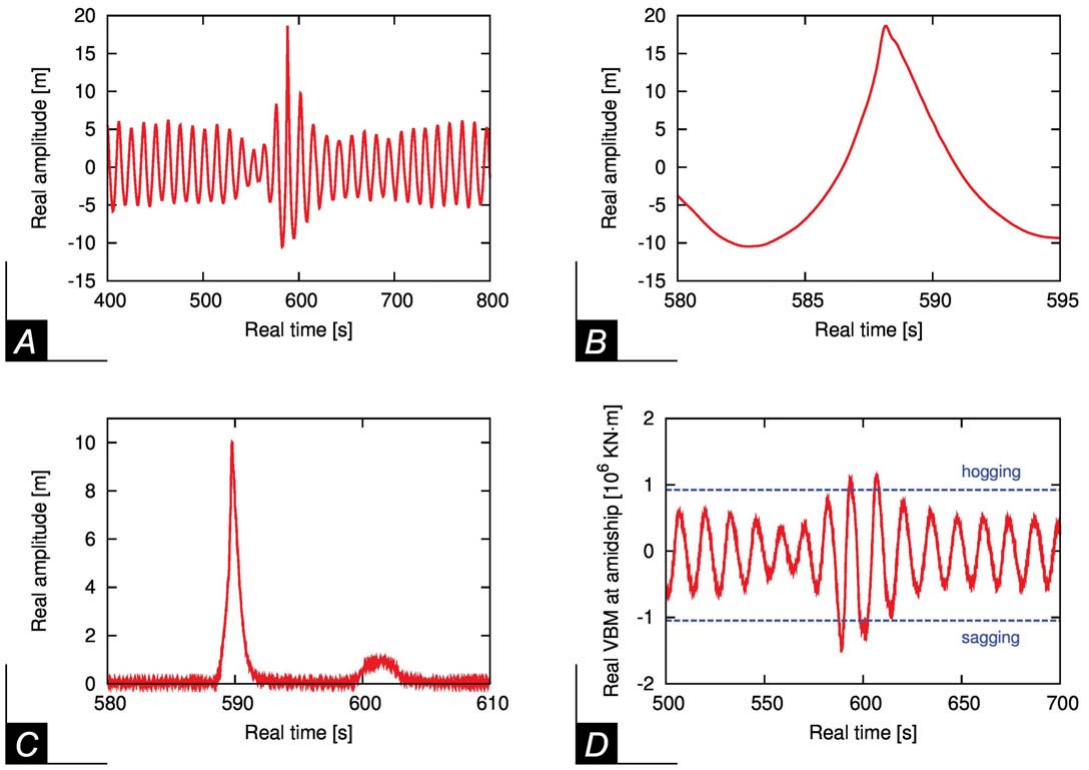
Quantitative results during the impact with a Peregrine breather. (A) The surface elevation just before impacting on the ship. (B) A detail of the surface elevation showing the steepest wave. (C) Height of water on deck of the ship as a function of time. (D) Vertical bending moment at amidship. The dashed lines denote the design vertical wave bending moment to be considered at an early stage.

Interesting quantitative measures during this sea-keeping experiment are presented in Figure 4. The nonlinear focusing mechanism responsible to the rogue wave event is clearly present observing the surface elevation dynamics in front of the ship (Figures 4A and 4B). The water gauge placed on deck measured a water column corresponding at 10 m full scale and the force transducer registered an extraordinarily large vertical bending moment (vbm) during the impact (Figures 4C and 4D respectively). The black dashed lines in Figure 4D illustrate additionally the design vertical wave bending moment to be considered at an early stage of the design process. The exceedance of this threshold is not resulting in an inevitable structural failure of the ship as additional parameters must be considered for the dimensioning of the transverse section but it shows that the wave is severe and a limiting case for the chemical tanker regarding the vertical bending moment. Moreover, the present results reveal that the impact of rogue waves on ships can be devastating: they can bring up a white wall of water as high as a three-storey building, spilling 2000 tons of water with a velocity of 70 km/h over the foredeck of the ship.

Concluding, Peregrine breathers have here been exploited for the first time to generate rogue wave events for the systematic investigation of the impact on ships. Whether these results will change the standards for building ships is a matter that should be investigated in the near future. However, the experimental generation of breather-type waves and its application to sea-keeping tests reveal that NLS breathers are not only beautiful mathematical objects but have an important impact in the study of wave/structure interaction during extreme ocean conditions.

## Supporting Information

Movie S1 A complete movie showing the experiments done at the Technical University of Berlin. Different breathers hitting the chemical tanker varying the ratio between the tanker length and the carrier wave length are shown.
(M4V)
